# Amplitude Analysis of High-Rate GNSS Measurements in the Frequency Domain

**DOI:** 10.3390/s26072025

**Published:** 2026-03-24

**Authors:** Caroline Schönberger, Werner Lienhart

**Affiliations:** Institute of Engineering Geodesy and Measurement Systems, Graz University of Technology, Steyrergasse 30, 8010 Graz, Austria

**Keywords:** GNSS, global navigation satellite system, vibration monitoring, amplitude, damping

## Abstract

The need for Structural Health Monitoring is evident in order to ensure the safety of civil infrastructure. The goal of vibration monitoring is to derive the eigenfrequencies, mode shapes and damping of a structure. A change in the eigenfrequency over time can indicate deterioration or damage in a structure. The amplitude can be used to calculate the damping ratio. As the damping ratio is amplitude-dependent, it is important to correctly determine the amplitude values. This study focuses on the amplitude correctness of high-rate Global Navigation Satellite System (GNSS) receiver data. In an experiment with controlled oscillations with a shaker and a Laser Triangulation Sensor (LTS) as a reference, the vibration amplitudes derived by GNSS measurements were analyzed, using time-frequency techniques like Short Time Fourier Transform (STFT) and Wavelet Transform (WT). We demonstrate that vibrations in the millimeter range can be derived from the measurements of satellites orbiting 20,000 km above Earth. However, the amplitudes of the determined frequencies show systematic errors up to 60% when compared to independent reference measurements. We introduce a correction method to reduce this error by applying a frequency-dependent correction function.

## 1. Introduction

To ensure the safe operation of civil infrastructure, monitoring systems are applied to detect damages and to prevent potential failures. Vibration monitoring is one possibility of structural monitoring, where damage can be detected that is not yet visible. Therefore, sensors are mounted on a structure to collect vibration data in the time domain. The data time series is then transferred into the frequency domain to compute the dynamic response of a structure. As a result, modal parameters such as frequency, mode shape and damping are determined and used as damage indicators [1].

Several methods are available for estimating damping from ambient vibration measurements, either for individual events or continuous records. One commonly used approach is the curve-fitting (or adjustment) method, in which damping is determined by fitting an exponential decay function to the amplitude envelope of the measured oscillatory response and extracting the corresponding decay rate; see Equation (1). This method is based on the equation of a damped harmonic motion, where the initial amplitude $A_{0}$ (at *t* = 0) decays over time according to the exponential factor $e^{-\xi \omega_{1}t}$. Here, $\omega_{1}$ denotes the measured damped circular frequency, *ξ* represents the modal damping ratio, and $\phi_{1}$ is the phase at *t* = 0 [2].

(1) $$x(t)=A_{0}\;e^{-\xi \omega_{1}t}\;cos(\omega_{1}t-\phi_{1})$$

Another widely used approach is the logarithmic decrement method, in which damping is estimated from the natural logarithm of the ratio between successive peak amplitudes of the oscillatory response; see Equation (2). In this expression, n denotes the number of cycles, while $x_{q}$ and $x_{q+n}$ represent the corresponding maximum displacements [3].

(2) $$\xi =\frac{ln(x_{q}/x_{q+n})}{2\;\pi \;n}$$

Other common methods focus on the frequency characteristics of a damped system, such as the bandwidth method, the half-power bandwidth method, or the stochastic subspace identification [4].

**Figure 1:**
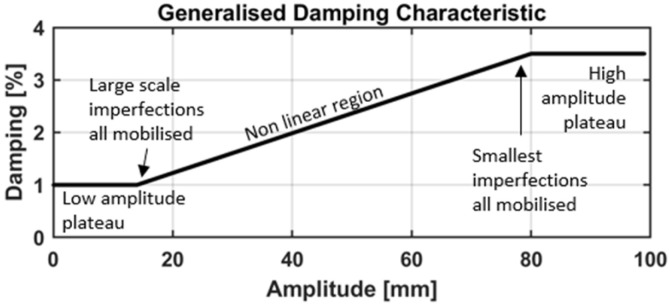
Generalized damping characteristic for structures, based on [5].

The previously presented equations provide common approaches for estimating structural damping. A scaling error in the amplitudes has no effect on the calculations because they depend only on the amplitude ratios. However, observations from measurements of real structures indicate that the effective damping can depend on the vibration amplitude, with larger response amplitudes generally leading to increased energy dissipation. This behavior has been demonstrated through analyses of the full-scale measurements of civil engineering structures, which show that the damping ratio tends to increase as the vibration amplitude grows. To account for this effect, an amplitude-dependent nonlinear damping representation can be used, in which the effective damping ratio is expressed as in Equation (3):

(3) $$\xi_{A}=\xi_{0}+\xi_{I}*A$$

where $\xi_{A}$ is the effective damping ratio at response amplitude A, $\xi_{0}$ represents the small-amplitude damping, and $\xi_{I}$ describes the rate at which damping increases with amplitude. The resulting relationship between damping and the vibration amplitude is illustrated by the generalized damping characteristics in Figure 1 [5].

In structural monitoring, most signals are time-varying and non-stationary. Therefore time-frequency techniques such as Short Time Fourier Transform (STFT), Wavelet Transform (WT) or Hilbert transform (HT) with their modifications are available [6,7].

Common measurements for vibration monitoring are accelerations, using accelerometers [8,9,10], and strain or strain rates, using fiber optic sensing systems [11,12,13]. Due to the increase in the data rate for Global Navigation Satellite System (GNSS) receivers up to 100 Hz, new opportunities arise which have been explored in laboratories [14,15,16,17,18,19,20,21] and on bridges [22,23,24], chimneys [25] and high-rise buildings [26] in recent years. It is shown that the computation of eigenfrequencies is effective in GNSS measurements.

Modern GNSS receivers are typically capable of capturing raw data and coordinates at 20 Hz or more. This is well suited for capturing the eigenfrequencies of civil structures as, for long bridges, towers, and tall buildings, the harmonic frequencies typically range from 0.1 Hz to 10 Hz [27]. A closer look at the expected eigenfrequencies of bridges shows frequencies between 1 Hz and 10 Hz for span lengths from 20 m to 100 m [28] and between 0.4 Hz and 6.5 Hz for buildings with more than four stories [29].

In order to measure the dynamic GNSS performance, controlled vibration experiments with shaking tables have already been initiated, as listed in Table 1, and many mention amplitude errors without further elaboration. As an example, in [18], the amplitudes of 5 mm, 10 mm and 15 mm were tested at the frequencies of 1 Hz, 2 Hz and 4 Hz, and an amplitude error of 53–76% at 2 Hz was reported. Refs. [14,15,16,17,18,19] show differences in the amplitude for a certain frequency with a certain amplitude.

One of the shortcomings of these studies is that all experiments were carried out only in a horizontal direction. Furthermore, compared to the experiments described in [14,15,16,17,18,19], the investigations in this paper:

- Focus on vertical vibrations, which are highly important for many civil structures, e.g., bridges.
- Give a more complete setup, including more receivers and more tested frequencies, amplitudes, and frequency sweeps. Overall, we cover amplitudes from 1 mm to 10 mm over a frequency range of 1 Hz to 8.6 Hz.
- Detect the frequency-dependent behavior of the amplitude errors.
- Introduce a correction function to correct the amplitude errors.

## 2. Experimental Setup

**Figure 2:**
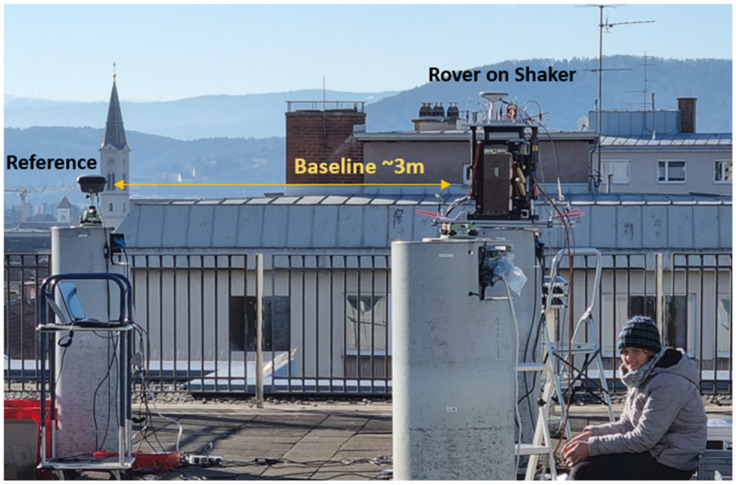
Overview of the experimental setup at the rooftop laboratory at Graz University of Technology.

This research is based on high-rate GNSS data collected at the rooftop laboratory at Graz University of Technology, Austria, as shown in Figure 2, on 17th, 18th and 20th January 2022 from 12 p.m. to 5 p.m., with similar weather conditions. The receivers collected phase measurements every 0.05 s (data rate of 20 Hz), which is sufficient for the determination of the first eigenfrequencies of many civil structures, considering the Nyquist–Shannon theorem [30]. Various high-end GNSS receivers were mounted, one after the other, on a shaking table. The shaking table was operated in horizontal and vertical directions, with a schedule including frequency sweeps from 10 Hz to 0 Hz and constant oscillations for 60 s with a pre-defined frequency and different amplitudes. A local reference station at 20 Hz was mounted next to the shaker, with a baseline distance of approximately 3 m. With this reference station, double differences (DDs) can be calculated between a pair of satellites and the two receivers. This eliminates clock errors from both the satellite and the receiver. Furthermore, orbit and tropospheric errors are almost eliminated or at least significantly reduced at such a very short baseline [31].

For GNSS data analysis, GPS and Galileo signals were used, based on the considerations in [32]. The data were processed kinematically, combining forward and backward solutions, in RTKLIB 2.4.3 [33]. The further core parameters are an elevation mask of 10° and a fix-and-hold integer ambiguity resolution mode. IGS antenna correction files were applied for phase center corrections. The resulting geographic φ and λ coordinates are then transformed into a local plane coordinate system. Depending on the orientation of the shaker, a 1D time series is taken for data analysis. In MATLAB R2018a [34], an outlier detection based on the median in a 10 s moving window was performed. Next, the data were high-pass filtered using a 2nd order Butterworth high-pass filter with an edge frequency of 0.5 Hz. This frequency was chosen because all tested excitation frequencies were higher than 1 Hz. Thus, the analysis band lies well above the cutoff frequency, and we did not observe measurable attenuation within the band of interest. The entire setup was designed to resemble the typical dynamic movements of bridges and buildings.

For amplitude analysis, STFT and WT are used. Both are widely applied in civil engineering. Details on these analysis methods and the used equipment are given in Section 3. More information on the experiment with the shaker and the results are described in Section 4.

## 3. Methods and Equipment

### 3.1. Frequency Analysis

The most commonly known method for analyzing a data time series for its frequency content is the Fourier Transform (FT), where the entire signal is transformed from the time domain into the frequency domain by computing the correlation of the signal with a pure sine wave; see Equation (4). As a result, the global frequency content F(s) of the entire signal f(t) is computed with equal weighting [35].

(4) $$F(s)=\int_{-\infty }^{\infty }e^{-ist}\;f(t)dt$$

Therefore, there is no temporal specificity, and the frequency and amplitude are averaged over time. Hence, FT is well suited for stationary signals that do not change over time [6]. Fast Fourier Transform (*FFT*) is an efficient algorithm for discrete (digital) data with an equal time spacing. The signal length N in seconds affects the frequency resolution Δf, with Δf=1/N, as well as the noise level in the frequency spectrum.

The peak amplitude *A* is computed via Equation (5) [36].

(5) $$A=\sqrt{{real[FFT(A)]}^{2}+{imag[FFT(A)]}^{2}}$$

To analyze data from Structural Health Monitoring (SHM) or data from our experimental setup, where the excitation is non-stationary or time-varying, a time-frequency analysis is necessary. In this work the STFT and the WT are applied, which are both based on the FT.

#### 3.1.1. Short Time Fourier Transform (STFT)

In STFT, a moving fixed-length window that shifts over the time axis of the signal is introduced. The signal within the window is then treated as a stationary signal, and an FT is computed. The result is highly dependent on the fixed window length (WL), which determines the fixed time-frequency resolution. There is a trade-off between the temporal resolution induced by the WL and the frequency resolution Δf [37].

**Figure 3:**
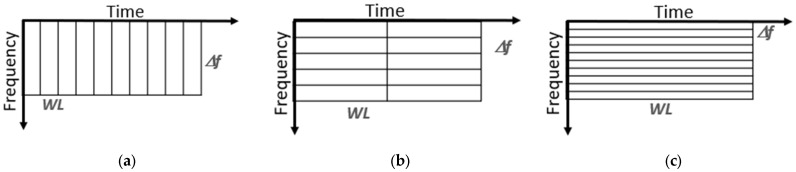
Schematic presentation of the STFT with various WLs. From (**a**–**c**), the temporal resolution decreases while the frequency resolution increases.

The exact Δf depends on the window function, with Δf∗WL ≥1/2 [38]. For a rectangular window, Δf=1/WL [36]. It is important to note that the frequency resolution depends only on the WL and not on the measurement frequency. For example, a rectangular window of one minute (60 s) results in a frequency resolution of 1/60 s = 0.0167 Hz. Figure 3 shows three time-frequency planes divided into rectangular windows of equal size. A short WL provides a good time resolution but does not identify different frequencies very well, whereas a long WL provides a good frequency resolution but makes it difficult to see the exact time of appearance. Therefore, it is not possible to achieve a high frequency resolution and a high time resolution simultaneously [6].

A known limitation of the rectangular window is its relatively high spectral leakage, which results from the abrupt discontinuities at the boundaries of each window. To reduce this effect, tapered windows, e.g., Hann and Hamming, are often used in spectral analysis. These windows significantly reduce spectral leakage but also broaden the main frequency lobe in the spectrum and hence reduce the frequency resolution [39].

The computation time of an STFT is fast [6], and it is commonly used in SHM [8,10].

In the present work, the rectangular window was selected to maintain a maximum frequency resolution and a straightforward segmentation scheme.

#### 3.1.2. Wavelet Transform (WT)

The main difference between Wavelet Transform (WT) and Fourier Transform (FT) lies in the way the signal is analyzed. While FT decomposes a signal into pure sine waves, for WT, $W_{f}(a,b)$ measures the correlation between the signal and a localized wavelet function ψ(t), as shown in Equation (6) [35]:

(6) $$W_{f}(a,b)=\frac{1}{\sqrt{\left|a\right|}}\;\int_{-\infty }^{\infty }f(t)\;\psi (\frac{t-b}{a})\;dt$$

**Figure 4:**
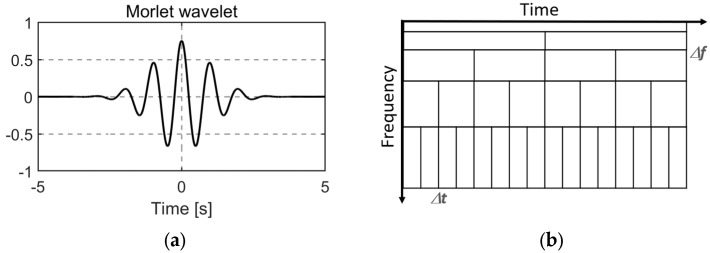
(**a**) Morlet mother wavelet; (**b**) multiresolution.

A wavelet, or mother wavelet, is a short oscillatory waveform designed to capture localized frequency content. Figure 4a illustrates the widely used Morlet wavelet, although numerous alternative mother wavelets exist [40].

A family of wavelets is then generated from the mother wavelet according to Equation (7) [35]:

(7) $$\psi_{a,b}(t)=\frac{1}{\sqrt{\left|a\right|}}\;\psi (\frac{t-b}{a})$$

This formulation preserves the wavelet’s time-frequency localization while allowing variations in position b and scale a, enabling a simultaneous analysis across both domains [41]. In the continuous Wavelet Transform (CWT), the signal is effectively convolved with this entire wavelet family.

The amplitude of the CWT is obtained from the magnitude of the complex coefficients, defined as Equation (8).

(8) $$A=\sqrt{{real[W_{f}(a,b)]}^{2}+{imag[W_{f}(a,b)]}^{2}}$$

As a result, a multiresolution analysis can be performed, where a signal is analyzed at different frequencies with different resolutions. In SHM, it is assumed that low frequencies persist for longer and that high frequencies occur sporadically. Therefore, the resolution can be chosen accordingly; see Figure 4b. The temporal resolution is the same as the time resolution of the input signal; therefore, a good temporal and frequency resolution can be achieved with WT [6].

### 3.2. Receivers

For this study, the following high-end receivers were used: Leica Geosystems AG, Heerbrugg, Switzerland, GS16, GS18, GR30, GMX902 and NovAtel Inc., Calgary, AB, Canada, PwrPak 7D. Table 2 summarizes the main parameters found in their data sheets. Some are designed for monitoring purposes (GMX902 and GR30) and others are mainly designed for surveying (GS16, GS18). The main purpose of the PwrPak receiver is robotics or autonomy due to its ability to record data from two antennas. GS16 and GS18 are ‘SmartAntennas’, which means that the receiver is integrated into the antenna, and for the others an external cable-connected antenna is needed. For these receivers, a Leica AS10 antenna was used. All used receivers are able to collect the GNSS code and phase measurements of the currently available satellite systems, including GPS, Galileo, GLONASS, and Beidou, with all included frequencies at a data rate of 20 Hz. The accuracies for kinematic post-processing with baselines less than 30 km are given for the Leica receivers in the data sheets, with 10 mm + 1 ppm (GMX902) and 8 mm + 1 ppm (GS16, GS18, GR30) in the horizontal direction and 20 mm + 1 ppm (GMX902) and 15 mm + 1 ppm (GS16, GS18, GR30) in the vertical direction. For PwrPak, this value is not given in the datasheets, but a value for the Real Time Kinematic (RTK) is available, at 1 cm + 1 ppm.

Data storage (Laptop, SD-card, USB) and data formats differ but can be converted into the Receiver INdependent EXchange format RINEX 3.02.

## 4. Experimental Results

**Figure 5:**
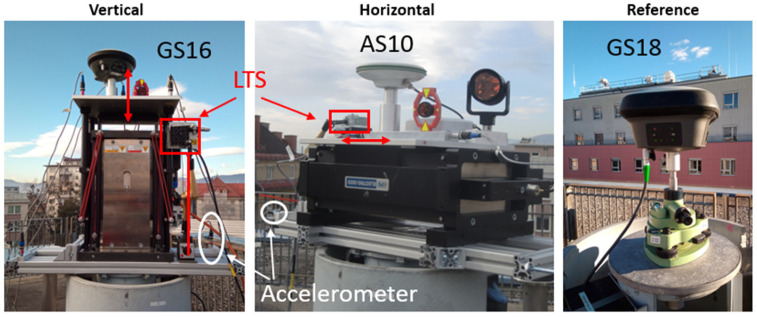
Experiment setup at the rooftop laboratory. Monitoring site with shaker in vertical (**left**) and horizontal (**center**) direction and the reference station (**right**).

The rooftop laboratory at Graz University of Technology is ideal for GNSS measurements since it provides an open view of the sky and the antennas can be mounted on stable surveying pillars. An overview of the laboratory is already shown in Figure 2. Detailed photos of the experiment with an APS Dynamics, Inc., San Juan Capistrano, CA, USA, 400 shaker [47] operated in vertical and horizontal directions are given in Figure 5. For the ground truth, a Laser Triangulation Sensor (LTS) was installed that followed the movement of the shaking table. The data rate of the used LTS Micro-Epsilon Messtechnik GmbH & Co. KG, Ortenburg, Germany, ILD-1700-50 is 312.5 Hz, and the measurement precision is 3 µm [48]. The measurement range is between 45 mm and 95 mm, enabling amplitudes of up to ±25 mm to be measured. The LTS was aligned parallel to the shaker’s movement axis. This was verified by checking that the laser spot of the LTS was in the same position on the shaking table at the minimum and maximum range positions of the LTS. This approach ensures a misalignment of less than 1 mm perpendicular to the shaking direction. Such a misalignment would result in a length error of less than 0.01 mm, which is negligible for the purposes of this investigation. Furthermore, the stability of the LTS mounting point and the pillar was checked using an accelerometer mounted on the frame on the top of the pillar. The reference antenna was mounted on a double-shielded surveying pillar. This protects the core of the pillar from direct sunlight, thus avoiding pillar bending due to one-sided sun illumination. Furthermore, the pillar is not directly exposed to wind. All experiments were carried out in low wind speeds, which was verified by a nearby weather station that recorded wind speeds of less than 4 m/s. Additional complementary sensors were accelerometers and a prism dynamically tracked with a Robotic Total Station (RTS). As these are not the focus of this paper, they are described in [49].

**Figure 6:**
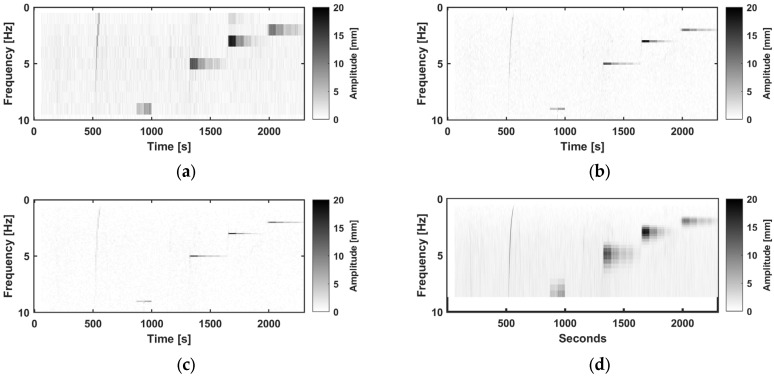
One test run with the shaker in vertical setup in the frequency-time domain using an STFT with WL. (**a**): 1 s; (**b**): 5 s; (**c**): 10 s. (**d**): WT recorded by PwrPak; frequency sweep (500–580 s), vibration at 9 Hz (870–1000 s), 5 Hz (1300–1600 s), 3 Hz (1600–1900 s), and 2 Hz (2000–2300 s).

Various frequencies and amplitudes up to 10 Hz and 20 mm were tested. A visualization of the GNSS results over the experiment using the PwrPak receiver in the vertical direction is shown in Figure 6, with an STFT with a WL of 1 s, 5 s and 10 s and a WT. The time-frequency dependency in the STFT and the multiresolution of the WT can be clearly seen. The standard deviation in the time domain of 5 min only noise datais 2.4 mm in the vertical direction, which corresponds to a noise level in the STFT of 1 mm (WL 1 s), 0.4 mm (WL 5 s) and 0.23 mm (10 s). In the horizontal direction, it is less than half, with 1.1 mm for the time series and 0.4 mm (WL 1 s), 0.2 mm (WL 5 s), and 0.15 mm (10 s).

No significant difference was found in the data recorded by different receivers; hence, data from all receivers are part of this paper for the different situations, namely amplitude steps and frequency sweeps in the horizontal and vertical directions.

### 4.1. Amplitude Steps

**Figure 7:**
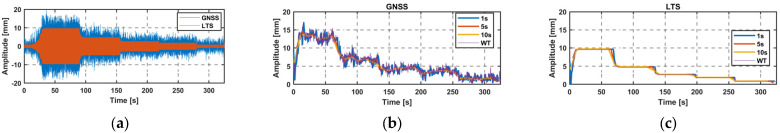
(**a**) Vertical time series for oscillations at 5 Hz and various amplitudes in the time domain for GNSS (PwrPak) and the LTS data; amplitudes derived using (**b**) GNSS and (**c**) LTS at 5 Hz after an STFT with WLs of 1 s, 5 s and 10 s and a WT.

Various amplitudes, up to 10 mm, were studied for a duration of ~60 s at frequencies of 2 Hz, 3 Hz and 5 Hz. Figure 7 shows the amplitude steps with a frequency of 5 Hz for the vertical setup and the PwrPak receiver in the time domain and after STFT and WT. In the time domain, shown in Figure 7a, it is already visible that the noise is higher for GNSS data than for LTS data. GNSS and The LTS amplitudes in the time-frequency domain, shown in Figure 7b,c, are internally consistent, but a difference can be clearly seen between the two instruments. In the GNSS data, a higher amplitude variation can also be seen in the time-frequency domain, with mean standard deviations summarized in Table 3.

**Figure 8:**
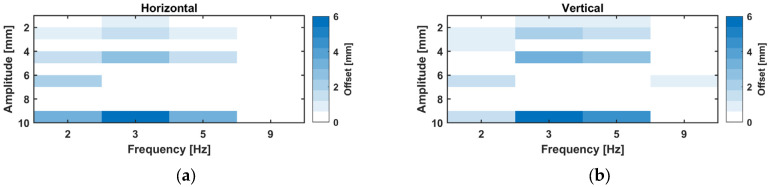
Mean difference between GNSS, (**a**) GR30, and (**b**) GMX902 and LTS-derived amplitudes.

For all tested frequency–amplitude combinations, a heat diagram, shown in Figure 8, was created by computing the mean difference in GNSS data versus LTS data using the results of the STFT.

These results are independent of the used WL. In addition to the amplitude steps at 2–5 Hz, an amplitude of ~6 mm was tested at 9 Hz in the vertical direction. It can be seen that, regardless of the size of the amplitude, the derived amplitude was too high for the GNSS data. The difference increased with the size of the amplitude, and, at a ~10 mm amplitude at 3 Hz, the difference was already ~6 mm.

**Figure 9:**
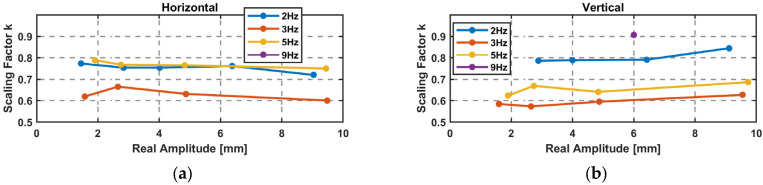
Scaling factor *k* for GNSS-derived amplitudes and a mean value per frequency: (**a**) GR30 and (**b**) GMX902.

The difference can also be represented as the scaling factor k[f] in Equation (9), where the real (LTS) amplitude is divided by the GNSS-derived amplitude. Each factor is graphically shown in Figure 9 for the amplitude steps. A frequency dependency of the scaling factor is clearly visible, which is similar for the horizontal and vertical direction. In addition, a mean value is given for each frequency in Table 4.

(9) $$k[f]=\frac{A_{real}}{A_{GNSS}}$$

With the scaling factor, a corrected amplitude can be computed using Equation (10).

(10) $$A_{\_corr}=k[f]*A_{\_GNSS}$$

Table 4 can serve as a look-up table for applying frequency-dependent corrections. In order to extend the correction function for the entire frequency range of 1 Hz to 9 Hz, further tests with frequency sweeps were performed.

### 4.2. Frequency Sweeps

**Figure 10:**
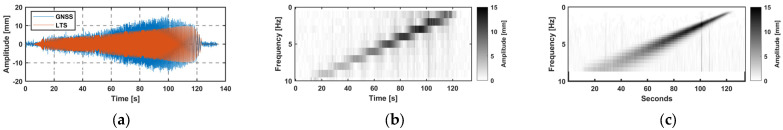
(**a**) Horizontal sweeps in the time domain for GNSS data collected with the GS16 and the LTS data; (**b**) GNSS data with an STFT WL of 1 s; (**c**) WT GNSS.

For this setup, the shaker started with a rather small amplitude at 10 Hz and reduced the frequency linearly or logarithmically to 0 Hz while increasing the amplitude. The linear sweep duration was around 2 min. With the application of an STFT with WLs of 1 s, 5 s and 10 s and a WT, the sweep is transformed into the time-frequency domain. For an STFT, a rather small WL has to be chosen due to the importance of the temporal resolution. Figure 10 shows the results for one linear sweep in the horizontal direction recorded by GS16 in the time domain (a) from GNSS and LTS data, and from GNSS data with an STFT WL of 1 s (b) and a WT (c).

**Figure 11:**
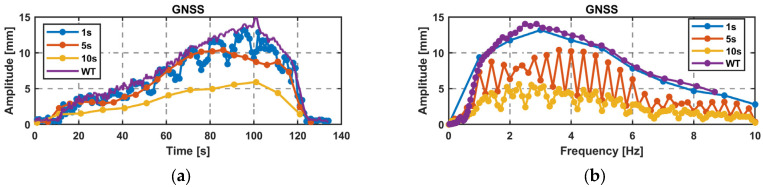
STFT WL of 1 s, 5 s, and 10 s and WT for GNSS data. (**a**) Amplitude over time; (**b**) amplitude over frequency.

Looking at the maximum amplitude over time (Figure 11a) and the maximum amplitude over frequency (Figure 11b) for the GNSS data, the limitations of STFT compared to WT are clearly evident. With a limited frequency resolution of 1 Hz for a WL of 1 s, the maximum amplitude moves up and down. This can be explained by spectral leakage, where the amplitudes fall into two frequency bins, when the real frequency is not an integer. A similar behavior can be seen for the amplitude over frequency for WL 5 s and 10 s, where the amplitude is split into two time bins. Computations with a WL of 5 s or 10 s show a maximum amplitude that is too low due to having multiple frequencies occurring within a single time step. Since it is a linear sweep, the WL of 1 s best fits to the WT due to its high temporal resolution and the linear frequency change, which can also be seen with just a few data points.

Overall, the results obtained with a WL of 1 s and WT show strong agreement, confirming the same amplitudes. In the case of a frequency sweep and fast changes in amplitude and frequency, the application of the WT is more suitable due to the large number of data points available, especially at smaller frequencies.

**Figure 12:**
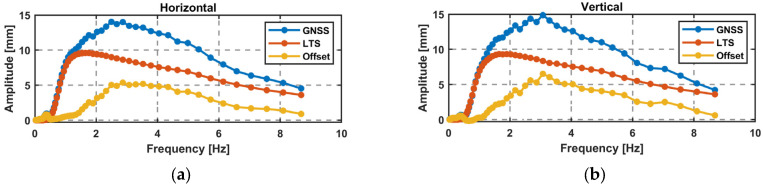
Difference in millimeter and in percent between the LTS and GNSS data for a linear sweep using the WT: (**a**) horizontal (GS16); (**b**) vertical (GR30).

In the vertical direction, another sweep was performed, and data were collected with the GR30 receiver. The results of the WT are used for a comparison of GNSS and LTS amplitudes. Firstly, the LTS data are downsampled to 20 Hz to achieve the same frequency resolution. The amplitude over the frequency is shown in Figure 12, with similar results for the horizontal sweep and the vertical sweep, where the GNSS amplitude has a positive offset compared to the LTS data.

**Figure 13:**
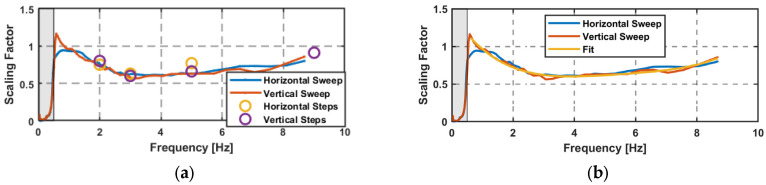
(**a**) Scaling factor for the sweeps and the steps; high-pass filter, 0.5 Hz; (**b**) approximated amplitude correction function.

The advantage of the sweep is that the scaling factor can be calculated continuously over the derived frequencies starting from the edge frequency of the high-pass filter at 0.5 Hz. Figure 13a displays the scaling factors over the sweeps and the amplitude steps, showing a good alignment. The scaling factor *k*(*f*) in the horizontal and vertical directions can be approximated by a fourth-degree polynomial; see Equation (11). With this polynomial, a corrected amplitude can be computed using Equation (12) over the frequencies of 1 Hz to 8.6 Hz. This is shown in Figure 13b with the scaling factors of the sweeps.

(11) $$k(f)=0.0009f^{4}-0.0198f^{3}+0.169f^{2}-0.627f+1.44\;\;f\in [1,8.6]Hz$$

(12) $$A_{\_corr}=k(f)*A_{\_GNSS}$$

### 4.3. Validation

**Figure 14:**
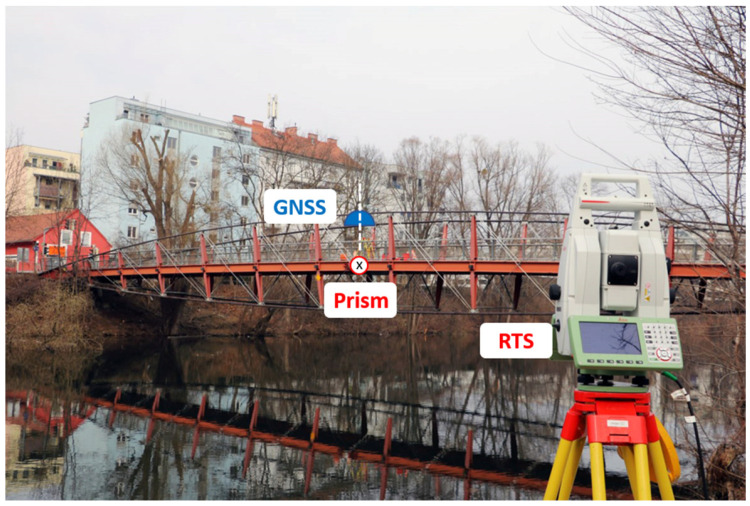
Overview of the setup at a nearby footbridge.

To validate the results, data collected at a nearby footbridge were taken, and GNSS-derived amplitudes were compared to the amplitudes derived using RTS measurements. The STD of the time series of the RTS measurements is 0.15 mm [47]. The measurement setup, with a GNSS receiver and a prism mounted on the bridge and an RTS placed at a nearby riverbank (~50 m distance), is shown in Figure 14. The GNSS antenna and the prism are placed in the middle of the bridge. The GNSS antenna is placed on a tripod, whereas the prism is mounted on the outer side with a direct line of sight to the RTS. Both sensors are leveled using a circular spirit level. Although the prism and the GNSS antenna are mounted in different places, their amplitude is comparable because only the Up component of the resulting 3D time series from both sensors is used.

A local GNSS reference station was placed within a distance of 200 m, with an open view to the sky. The bridge was excited by people walking or jumping on it.

**Figure 15:**
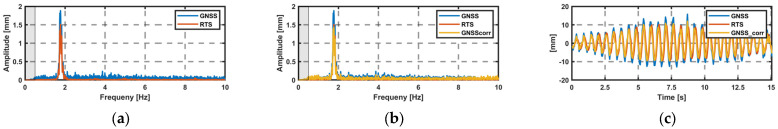
An FFT of GNSS and RTS data. (**a**) Original data; (**b**) additional corrected GNSS data is shown; (**c**) the scaling factor *k* = 0.76 of 1.77 Hz is applied on the data time series.

The recorded 20 Hz GNSS data were treated the same way as for the experiment in the laboratory, where GPS and Galileo signals were processed using RTKLIB. With the RTS, the 3D displacements of the prism could be computed at 20 Hz. Therefore, the displacements of the bridge in millimeters at a data rate of 20 Hz were available for both instruments. Further processing was done in MATLAB for both Up time series, and a high-pass filter and FFT were computed. For a time period of 100 s with strong excitation, the main frequency peak was calculated as 1.77 Hz for both instruments. A significant difference of 0.37 mm for the amplitudes at the main peak is visible in the frequency spectrum; see Figure 15a. A corrected GNSS amplitude was calculated by applying Equation (12) to the GNSS-derived frequency spectrum. According to Equation (12), *k* = 0.76 for *f* = 1.77 Hz, and, when applied to the data series, the difference in the main peak can be reduced to −0.08 mm, as shown in Figure 15b. This significant improvement can also be seen in the time domain, as shown in Figure 15c.

## 5. Discussion and Conclusions

This paper focused on the amplitude error of GNSS measurements for controlled oscillations in the frequency domain. In particular, the oscillations of frequencies from 2 to 5 Hz were tested with various amplitudes and frequency sweeps from 0 to 10 Hz. All tests were carried out with amplitudes below 10 mm. Remarkably, amplitudes of only a few millimeters could be derived from measurements from GNSS satellites orbiting 20,000 km above Earth.

In order to transform the time series into the time-frequency domain, an STFT with various WLs and a WT was performed. The clear advantages of the WT could be seen for the frequency sweeps, with a good temporal and frequency resolution. For amplitude steps where a frequency was held for a longer time and temporal resolution is not important, the STFT is preferred due to its better frequency resolution.

The results of the analysis on amplitudes are independent of the tested high-end receivers, the used analysis methods (STFT, WT), and the orientation of the oscillations (vertical, horizontal). The GNSS-derived amplitudes were too high, with a systematic frequency-dependent error. This error was identified as a scaling factor, which needs to be applied to the GNSS-derived amplitudes. With data from the sweeps, scaling factors are available over the entire frequency spectrum, and an amplitude correction function was determined.

This amplitude correction function was tested on GNSS data from a footbridge, which were compared to RTS data. For regular pedestrian traffic, vibrations in the millimeter range occur at this specific bridge. Without the correction function, the GNSS amplitude was again too high. After applying the amplitude correction function, the amplitude of the main peak was comparable.

The results presented here are results based on a few data sets for GNSS receivers with a data rate of 20 Hz. The scaling factors derived from the sweep and the frequency steps are highly reproducible. However, additional research on receivers with higher data rates, other RTK processing software, and more tested frequencies for longer durations is necessary to generalize the derived correction function.

In the context of structural monitoring, eigenfrequencies below 10 Hz are very common. An incorrect amplitude calculation can influence the correct interpretation of damping ratios. Therefore, this study is important for correctly interpreting GNSS vibration monitoring data.

## Figures and Tables

**Table 1:** Existing investigations on GNSS vibration measurements with shakers.

Year of Measurement	Data Rate Rover	Frequency [Hz]	Amplitudes [mm]	Relevant Output	
N/A	100 Hz	3.8	1.5, 6, 9	Amplitudes were too high	[14]
2019	20 Hz	0.8, 3.4, 7.6	5, 10	Small differences in the amplitudes, ranging from 0.1 to 0.6 mm	[15]
2022	100 Hz	0.11, 0.34	1, 2.7	Slight differences in the derived amplitudes	[16]
N/A	20 Hz	0.3–3	5, 10	Differences in the amplitude of less than 0.5 mm	[17]
2022	20 Hz	1, 2, 4	5, 10, 15	Amplitude error of 53–76% at 2 Hz	[18]
2023	20 Hz	0.88	15.1	Differences in the detected amplitude	[19]

**Table 2:** Main parameters of GNSS instruments found in the data sheets [42,43,44,45,46].

	GS16	GS18	GR30	GMX902	PwrPak 7D
**Horizontal accuracy**	8 mm + 1 ppm			10 mm + 1 ppm	10 mm + 1 ppm
**Vertical accuracy**	15 mm + 1 ppm			20 mm + 1 ppm	
**Maximum position update rate**	20 Hz				100 Hz
**Velocity accuracy**			Hz: 0.003 m/s V: 0.005 m/s		Hz: <0.03 m/s
**Typical application**	Survey rover		CORS station	Structural monitoring	Robotics/autonomy
**Antenna**	Internal		External AS10 antenna	Internal	External AS10 antenna

**Table 3:** Mean standard deviation per frequency–amplitude step using four different time-frequency analyses.

Time-Frequency Analysis	Mean STD (GNSS)	Mean STD (LTS)
STFT WL 1 s	0.5 mm	0.100 mm
STFT WL 5 s	0.3 mm	0.020 mm
STFT WL 10 s	0.2 mm	0.005 mm
WT	0.8 mm	0.003 mm

**Table 4:** Mean scaling factors for certain frequencies.

Frequency	k[f]_Horizontal	k[f]_Vertical
2 Hz	0.75	0.80
3 Hz	0.63	0.60
5 Hz	0.77	0.66
9 Hz		0.91

## Data Availability

The datasets presented in this article are not readily available because, the data are part of an ongoing study. Requests to access the datasets should be directed to c.schoenberger@tugraz.at.
